# Identifying unmet information needs of advanced cancer patients in Iran: An in‐depth qualitative study

**DOI:** 10.1002/hsr2.914

**Published:** 2022-10-29

**Authors:** Parasto Amiri, Ali Mohammadi, Kambiz Bahaadinbeigy, Behjat Kalantari Khandani, Vahid Maazed

**Affiliations:** ^1^ Medical Informatics Research Center, Institute for Futures Studies in Health Kerman University of Medical Sciences Kerman Iran; ^2^ Department of Health Information Technology, Paramedical School Kermanshah University of Medical Sciences Kermanshah Iran; ^3^ Medical Informatics Research Center, Institute for Futures Studies in Health Kerman University of Medical Sciences Kerman Iran; ^4^ Department of Oncology, Shahid Bahonar Hospital, School of Medicine Kerman University of Medical Sciences Kerman Iran; ^5^ Hematology and Oncology, Faculty of Medicine Kerman University of Medical Sciences Kerman Iran

**Keywords:** cancer, qualitative study, self‐care, unmet information needs

## Abstract

**Background and Aims:**

One of the main vital needs for self‐care in patients with advanced cancer is information need. Meeting this need has significant positive effects on improving their treatment and care. This study was conducted to identify the unmet information needs of patients with advanced cancer in Iran.

**Methods:**

This exploratory study was performed from July to February 2021 in the Kerman University of Medical Sciences cancer treatment centers. Oncologists selected eligible patients by purposeful sampling method. Semistructured and in‐depth interviews were conducted with selected patients to collect data. Interviews continued until data saturation. Each interview was audio‐recorded and transcribed verbatim.

**Results:**

In the interviews, 15 patients with advanced cancer ranging in age from 43 to 65 years participated. The most common type of cancer in women was breast (71.4%) and prostate (50%) in men. The two main categories of “types of unmet information needs” and “reasons for not meeting information needs” were extracted from the analysis of patient interviews, with six and four subcategories, respectively.

**Conclusion:**

Cancer patients had a large number of unmet information needs. At the time of identifying the unmet information needs of cancer patients, the basic reasons for not meeting these needs should also be considered because cultural differences and social gaps in societies are inevitable.

## 1 BACKGROUND

One of the most common causes of death worldwide is noncommunicable diseases such as cancer.^1^ Over the years, the spread of cancer in some countries has reached such a level that after cardiovascular disease, cancer is the leading cause of death among noncommunicable diseases.^2^, ^3^ Among the most deadly cancers worldwide are lung, breast, and colorectal cancers.^1^ According to the International Cancer Agency, in 2018, 18 million people worldwide were diagnosed with cancer annually, and 6 million died from the disease.^1^ Cancer has been the third leading cause of death in the Middle East in recent years.^4^ After cardiovascular diseases and road accidents, cancer has been the main cause of death in Iran.^5^, ^6^ In addition to the risk of death, cancer patients, especially in their advanced stages, face psychological problems such as fear, shock, anxiety, and physical problems such as pain from chemotherapy.^7^, ^8^, ^9^ Therefore, these patients must obtain more information to deal with the progression of the disease and the resulting problems.

In the advanced stages of cancer, patients often need more self‐care information for various reasons, such as tumor growth and side effects from various treatments such as surgery, chemotherapy, and radiotherapy.^10^, ^11^, ^12^ Increasing self‐care information in them will lead to increased participation in the treatment selection process, increase self‐care, reduce anxiety and worry, and thus improve quality of life.^13^, ^14^, ^15^, ^16^ Over the past few decades, extensive research efforts have been made to assess the information needs of patients with lung cancer, breast cancer, colorectal cancer, and various types of cancer.^17^, ^18^, ^19^, ^20^ The results of these studies suggest that patients often experience more complex symptoms and issues as cancer progress than in the early stages of the disease. The information needs of patients with advanced cancer have often been reported as unmet care needs.^21^

According to our research, most of the research has examined the information needs of cancer patients in western countries.^20^, ^22^, ^23^ The number of studies in this field is small in Asia, especially in Iran.^24^, ^25^ Due to Iran's cultural differences from western countries, this research may not be able to fully cover the information needs of Iranian cancer patients. Because in western culture, studies often pay attention to the physical dimension of care and ignore its spiritual dimension.^26^ While in Iran, due to Islamic religious and spiritual ideas,^27^ these ideas may be harmful or vice versa useful. On the one hand, cancer patients in Iran may not clearly state their information needs to not worry about their family environment (the harmful dimension of these thoughts). On the other hand, these patients may overcome their stress and anxiety with prayer and mystery and more calmly acquire their information needs (useful dimension of these thoughts).

Also, the ability of cancer patients to take care of themselves is important because it considers the care needs of these patients. For this purpose, several studies using quantitative and qualitative approaches have identified the information needs of cancer patients.^28^, ^29^, ^30^ So far, several cross‐sectional survey studies have been conducted, which did not identify most of the unmet needs due to the limited number of questionnaire items.^21^, ^28^ As far as we know, the available evidence has not explained the needs of patients with advanced cancer in Iran. For this reason, our study used the qualitative method because qualitative studies can deeply describe unknown or little‐known phenomena from the perspective of people who experience them in different cultures.^31^ Qualitative research is one of the best methods for investigating human phenomena and evaluating different perspectives and perceptions because it is impossible to fully investigate human, cultural, and social dimensions and values using quantitative research.^32^ In addition to the above, the increasing incidence of various types of cancer reinforces the need to meet the information needed to control and combat cancer among these patients in Iran.^28^ Due to the multidimensionality, complexity and ambiguity in the information needs of these patients, the present qualitative study was conducted by examining the perspectives and experiences of patients with advanced cancer to answer the following questions:

1. What information is not provided to Iranian patients with advanced cancer?
2. What is the reason for not meeting the information needs of Iranian patients with advanced cancer?

## 2 METHODS

This exploratory study was performed using the qualitative content analysis method. This study conducted interviews with cancer patients from July to February 2021 in the cancer treatment centers of Kerman University of Medical Sciences (KMUS) to collect data. Inclusion criteria include at least one type of cancer, advanced cancer (Stage III or IV), chemotherapy or radiotherapy, or both, over 18 years, ability to speak and understand Persian, and having skills in verbal communication was sufficient. Eligible patients were selected and introduced by purposive sampling by two physicians (oncologists).

The research team developed and approved the interview questions framework (two medical informatics specialists and two oncologists). Participating patients were asked questions about the information needed for self‐care so that they could share their experiences of ambiguities and issues with self‐care during illness. Interview questions included “What problems did you have as the disease progressed?,” “What information did you need to take care of yourself during chemotherapy or radiotherapy?” and “What did you do to resolve the problems?”

Due to the prevalence of COVID‐19 and quarantine restrictions, each eligible participant was interviewed semistructured and in‐depth through social media (WhatsApp video call) by the first author. However, before the start of each interview, a brief explanation about the subject and purpose of the research was provided to the patient to satisfy the patient to conduct the interview. Then, the electronic informed consent form designed by the research members was sent to the patient through social media (WhatsApp). The consent form emphasized the patient's consent to record his/her statements by the research members, the confidentiality of the recorded information and the right not to cooperate at any time during the research without giving a reason. If each patient agreed, the interview was conducted at the preferred time announced by him/her. During the interview, the patient's speech was recorded and transcribed. The average duration of each interview was 40 min (30–50 min). Data saturation took place in the 15th interview.

**Figure 1:**
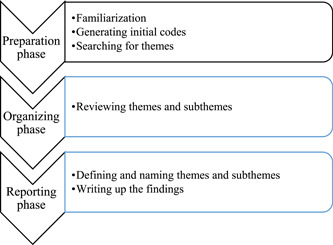
Data flow diagram of data analysis process

Data were analyzed using the content analysis method.^33^ Copies were uploaded in Atlas.ti8 software and coded by two members of the research. Qualitative content analysis was performed in four stages: “initialization,” “construction,” “rectification,” and “finalization.” In the “initialization” stage, two research members read the transcribed data many times to familiarize themselves with the data. In the “construction” stage, the primary codes were classified and compared according to their similarities and differences, and then they were assigned to different groups based on the research questions. In the “rectification” stage, most categories and subcategories were corrected by re‐evaluation. In the “finalization” stage, the final report was extracted from the analyses. A summary of the steps performed in this analysis is shown in Figure 1. Categories and subcategories were modified through three online sessions with an average time of 30 min.

The study was approved by the ethics committee of KMUS with code ethics IR.KMU.REC.1399.026. All participants filled out informed consent before the interview. To maintain the confidentiality and anonymity of the patient, before analyzing each interview, a unique code replaced the patient's name. This study has been reported in accordance with the Standard Qualitative Research Reporting (SRQR) guidelines (see Supporting Information: File 1). All methods were performed in accordance with the relevant guidelines and regulations of the Declaration of Helsinki.

## 3 RESULTS

The characteristics of the participants are presented in Table 1. The number of participating patients was almost equal in terms of gender. The average age of men and women was equal (approximately 52 years), and they believed in Islam. The most common type of cancer in women was breast (71.4%) and prostate (50%) in men. Most of them were married.

**Table 1:** Characteristics of patients participating in the interviews

Patients	Gender	Age	Occupational status	Religion	Cancer type	Economic status	Educational level	Marital status	Numbers of children	Cancer stage	Chemotherapy	Radiotherapy
P1	Male	43	Working	Muslim	Lung	Middle	Diploma	Married	5	IV	*	
P2	Male	51	Pensioners	Muslim	Colorectal	Low	High school	Married	4	III	*	*
P3	Male	56	Widowed	Muslim	Lung	Middle	Diploma	Separated	6	III	*	*
P4	Female	49	Working	Muslim	Breast	Middle	Associate degree	Divorced	3	IV	*	
P5	Female	43	Out of work	Muslim	Colorectal	Middle	Primary school	Married	4	IV	*	*
P6	Female	63	Out of work	Muslim	Lung	High	Primary school	Separated	7	IV	*	
P7	Male	54	Working	Muslim	Prostate	High	Associate degree	Married	5	III	*	
P8	Male	45	Working	Muslim	Lung	High	Diploma	Separated	5	III	*	
P9	Female	55	Pensioners	Muslim	Breast	Low	High school	Married	4	III	*	*
P10	Male	43	Working	Muslim	Prostate	Middle	Bachelor's degree	Married	3	IV	*	*
P11	Female	49	Working	Muslim	Breast	High	Diploma	Married	3	III	*	
P12	Male	60	Out of work	Muslim	Prostate	Low	Diploma	Married	5	IV	*	*
P13	Female	51	Out of work	Muslim	Breast	Middle	Associate degree	Divorced	8	IV	*	
P14	Female	54	Pensioners	Muslim	Breast	High	High school	Married	5	III	*	
P15	Male	65	Out of work	Muslim	Prostate	Middle	Diploma	Married	7	IV	*	

The codes obtained from the content analysis results were 243 initial codes; after deleting duplicates and similar items, 132 initial codes remained. Finally, these codes were covered in two main categories: “Types of unmet information needs” and “Reasons for not meeting information needs,” with six and four subcategories, respectively. The categories and their subcategories are shown in Table 2. Some exemplary quotes, indicated with (Q#) within the text, are available in Table 2.

**Table 2:** Summary of categories and subcategories resulting from the qualitative content of the interviews with exemplary quotes

Categories	Subcategories		Exemplary quotes
Types of unmet information needs	Information related to the nature of the disease		Q1: “I tried hard to find out more about cancer, but I could not find enough sources. I searched a lot in virtual networks and internet sites, but I could not get much information… Understanding the different stages {cancer} is also important to me to see what is going to happen to me…” (*P3, Male, Lung Cancer, Stage III*) Q2: “I needed someone to explain to me what this disease {cancer} is? What makes it happen? What causes it to recur? Could it involve another part of my body?” (*P10, Male, Prostate Cancer, Stage IV*)
	Information needs related to treatment methods		Q3: “This disease is like a giant to me that I am very afraid of… Some methods {treatment} that my doctor tells me very little, the risk is high… I'm afraid my body will not tolerate… I do not want to die so soon” (*P5, Female, Intestinal Cancer, Stage IV*) Q4: “The cost of treatment is very important to me… That's why whenever the doctor wants to prescribe a treatment for me, I first ask about the cost… I do not like my wife and children to be in trouble because of my illness” (*P1, Male, Lung Cancer, Stage IV*)
	Nutrition‐related information needs		Q5: “Getting information about nutrition and coping with reducing nausea and vomiting during chemotherapy is very important to me” (*P15, Male, Prostate Cancer, Stage IV*) Q6: “Due to loss of appetite, cancer patients may lose the nutrients their bodies need and this may cause their death… Being under the supervision of a nutritionist can prevent this from happening” (*P8, Male, Lung Cancer, Stage III*)
	Information related to the management of physical symptoms		Q7: “Chemotherapy is very painful. Although my pain tolerance is high… The pain is unbearable for me. When I undergo chemotherapy, it's like a bomb explodes in my body, it's really horrible…” (*P11, Female, Breast Cancer, Stage III*) Q8: “A particle that I work on my left hand becomes very swollen, It hurts a lot under my arms, It hurts so much that I feel like it wants to be torn” (*P9, Female, Breast Cancer, Stage III*)
	Information related to psychological support		Q9: “Ever since I suffered the pain of chemotherapy, I have a special fear of going to the hospital… Terrible cancer, I'm very, very scared of it. Not knowing about it makes it even scarier” (*P14, Female, Breast Cancer, Stage III*) Q10: “The most important thing for health is happiness, which I no longer feel in myself. I go for a walk in the park every morning, I do my shopping, and sometimes I go to a painting class in the afternoon. However, when I remember that the cancer giant has penetrated my body, I feel completely relaxed.” (*P6, Female, Lung Cancer, Stage IV*)
	Information related to financial support		Q11: “I am very upset about the insurance conditions. Insurance should help a lot more so that the patient does not spend so much. However, material conditions affect all aspects of life” (*P2, Male, Intestinal Cancer, Stage III*) Q12: “I leave the city for about eight hours every time I come for chemotherapy… The total cost of our bus ticket and food is… I have nothing {money} left…” (*P4, Female, Breast Cancer, Stage IV*)
Reasons for not meeting information needs	Patient	Low level of health knowledge	Q13: “I am only literate. I never received formal education about cancer. Sometimes I can not understand the words that the doctor tells me, unless she explains them to me many times.” (*P7, Male, Prostate Cancer, Stage III*)
		Undesirable physical condition	Q14: “I have had chemotherapy six times so far. Every time I get chemotherapy, I have severe vomiting that leaves me with my whole life. I no longer want to be on chemotherapy” (*P12, Male, Prostate Cancer, Stage IV*)
		Religious beliefs	Q15: “Every time I am hospitalized, I ask all those who know me to let me go. One does not know about her death” (*P5, female, bowel cancer, IV cancer*) Q16: “Ever since I got chemotherapy, I think more about that world and life after death. I went to the cleric of our local mosque many times and asked her my questions” (*P1, Male, Lung Cancer, IV Cancer*)
	Specialist	Lack of time	Q17: “My doctor does not have time to explain everything to me. He only advises me about medicines or daily needs.” (*P8, Male, Lung Cancer, Stage III*)
		Incomprehensible answers	Q18: “When I ask my doctor, she explains the information to me in detail. But because I do not understand some of his words, I can not fully understand his explanation” (*P13, Female, Breast Cancer, Stage IV*)
		Family and friends	Q19: “The compassion of those around me makes me more nervous, I have now come to terms with cancer, but I have heard many times from others that this man has cancer! Well, this is a disease like any other disease. Really, our people still do not know how to treat cancer” (*P2, Male, Intestinal Cancer, Stage III*)
		Community	Q20: “I do not want to sum up everyone, some are so short‐sighted that they look at us badly, of course, cancer is very bad in our society. Some look at us with pity. That's why I said that not everyone needs to know {I have cancer}” (*P9, Female, Breast Cancer, Stage III*)

### 3.1 Types of unmet information needs

Findings indicated patients' desire to receive more information about the nature, stages of progression, recurrence and metastasis of the disease, various treatments, nutrition, management of physical symptoms and pain, psychological support and treatment costs and insurance coverage.

#### 3.1.1 Information needs related to the nature of the disease

Most of the unmet information needs of the participants were information about the nature of the disease (14/15). Most of them did not know the factors that cause cancer. Many also stated that they did not have enough information about the nature, stages of progression, recurrence and metastasis of the disease and wanted to learn more (Q1, Q2).

#### 3.1.2 Information needs related to treatment methods

The interviews found that the participants were more familiar with different treatment methods through their physicians. Most patients (10/15) chose the appropriate treatment method based on survival rates and costs. Some patients' statements emphasize the importance of paying attention to this information need (Q3, Q4).

#### 3.1.3 Nutrition‐related information needs

All participants reported experiencing several annoying therapeutic side effects, such as loss of appetite, nausea, vomiting, changes in eating habits, and changes in taste (9/15). They wanted to learn more about nutrition. Some also found it necessary to consult a nutritionist for cancer patients. Q5 and Q6 indicate the importance of paying attention to nutrition in these patients.

#### 3.1.4 Information needs related to the management of physical symptoms

All patients in the present study had advanced cancer and received chemotherapy, radiotherapy, or both. These treatments led to physical side effects such as pain, hand swelling, decreased ability to perform daily tasks, indigestion and hair loss. Q7 and Q8 are examples of what some patients say during chemotherapy.

#### 3.1.5 Information needs related to psychological support

In this study, most people stated that the word cancer was a stressor for them (12/15). Participants stated that they needed more cancer information to maintain their mental health. Failure to pay attention to this need is a serious warning to reduce their quality of life. Q9 and Q10 are examples of the painful words of some patients.

#### 3.1.6 Information needs related to financial support

Participants in this study pointed to the inefficiency of health insurance coverage and the high cost of chemotherapy drugs (10/15). Most participants emphasized the need for financial support from government sources and the expansion of insurance coverage for medical expenses, especially chemotherapy drugs. For example, Q11 is a self‐explanatory part of what the patient says.

For patients in rural areas or patients living in areas far from the medical center, nonmedical expenses such as travel, accommodation, food, and so forth. can also be very problematic for patients and their families. Part of a patient's interview (Q12) demonstrates this need.

### 3.2 Reasons for not meeting information needs

Describing the experiences of study participants showed that the reasons for not meeting the information needs of patients are numerous and complex. Several factors (such as the patient, the treating physician, family or friends and the community) can lead to unmet these needs.

#### 3.2.1 Patient

##### Low level of health knowledge

One of the patients' concerns was that due to their low level of health knowledge, they could not establish a proper relationship with the medical staff during their treatment process (5/15). This leads to a small and sometimes misunderstanding of the information experts provide. It also limits their ability to search for health information (Q13).

##### Unfavorable physical condition

Some patients reported experiencing unpleasant side effects such as pain, nausea, and vomiting after receiving chemotherapy (6/15). Therefore, they were not physically good enough to seek information until they recovered from the side effects. This reason is stated in part of a patient interview (Q14).

##### Religious beliefs

Asking for forgiveness, asking for forgiveness from friends and acquaintances, was one of the mental occupations of cancer patients. This reduced their motivation to request information (11/15). This reason is stated in part of a patient interview (Q15).

Other reasons for not meeting information needs have been patients' mental preoccupations with death, the afterlife, and human destiny after death at the time of illness (8/15). These cases became more pronounced as the disease progressed and reduced their motivation to obtain information. Part of a patient's interview (Q16) explains this.

#### 3.2.2 Specialist

##### Lack of time

One of the main reasons cancer patients' information needs were not met was that health care professionals, due to a large number of their patients, are usually so busy that they cannot provide comprehensive and accurate information (4/15). Part of a patient's interview (Q17) explains this.

##### Incomprehensible answers

Another reason for not meeting cancer patients' information needs was physicians' vague responses to patients (3/15). Because doctors use specialized terms and expressions in their training that patients usually do not understand. This prevents the correct transfer of information to patients. Patients are also unable to understand physicians' responses due to low levels of health literacy, so physicians are content to provide only health information (Q18).

##### Family and friends

Family and friends are important in managing complex diseases such as cancer (8/15). Family psychological support and their appropriate response to cancer affect these patients' quality of treatment and life. Lack of psychological support discourages the patient's focus and ability to obtain more information about cancer (Q19).

##### Community

After being diagnosed with cancer, some patients limit their social interactions to avoid unpleasant encounters such as pity, heavy looks, and so forth. (6/15). This reduced their chances of getting information from others. Part of a patient's interview (Q20) explains this.

## 4 DISCUSSION

Promoting health and increasing patient self‐care largely depends on meeting their information needs. The first step in meeting these needs is to identify them. Therefore, this study aimed to identify the information needed by patients with advanced cancer. Patients with advanced cancer in this study experienced numerous unmet information needs. According to Hasson, cancer patients in the advanced stages of the disease need to receive more information about treatment and other services than cancer patients in the early stages.^34^ Analysis of patients' experiences participating in the present study led to the emergence of two categories: “types of unmet information needs” and “reasons for not meeting information needs.” The analysis of the findings indicates that the participants did not have sufficient and appropriate information in various fields such as the nature of the illness, treatment, mental, physical, nutritional, and financial. The cause of their unmet was also related to the patient, physician, family, and community.

Attention to meeting information needs is critical to cancer control.^35^ So far, several countries have identified and categorized these needs in cancer patients.^36^, ^37^, ^38^, ^39^ A systematic review study for advanced cancer patients categorized all information needs into 12 categories.^21^ The present study covered only 6 of the 12 categories. For example, in the present study, no patient indicated a need for sexual information. While 50% of the male participants in the present study had prostate cancer, we know that prostate cancer treatment significantly impairs male sexual function.^40^ The reason for this discrepancy may have been the lack of clear questions about sexual information needs in the present study. In the Boyes study,^37^ the questioner explicitly asked patients this question. Another reason may be found in the religion of the people of Iran (Islam) because modesty is emphasized in Islam.^41^, ^42^ Due to modesty, many Muslim patients in Iran are embarrassed to express their sexual problems during treatment.

In cancer patients, meeting information needs is one of the most important standards of care and support^34^ because having information plays a big role in patients' efforts to fight cancer.^43^ Patients who know enough about their disease (e.g., cancer) are better able to control their emotions about it.^43^ In our study, the most information needed among participants was related to the nature of the disease (such as the cause of the disease, how the disease progressed, and the recurrence of the disease). However, in the Boyes study,^37^ the highest support needed in cancer patients is self‐care to meet physical needs and help them adapt to the disease. In Mazhari and Khoshnood's study,^29^ the need for compassionate and knowledgeable caregivers was the most important need of patients. The reason for this discrepancy may depend on factors such as age, gender, and marital status, level of education, income level, ethnicity and type of cancer.^21^

In several studies, age, gender, marital status, education level, and income level were insignificantly associated with patients' unmet needs.^44^, ^45^, ^46^, ^47^, ^48^ A study in Iran also showed no significant difference between the health information needs of cancer patients with gender, age, marital status, education level, occupation, lifestyle, and rural–urban residence.^49^ In our study, more than 70% of all patients were married, half of whom were women. Since married patients have more responsibilities at home, they may have more unmet information needs than unmarried patients. A study in China confirms this.^44^ Also, with increasing age, the unmet needs of patients decrease.^48^ Many studies have also reported that female patients have more unmet physical needs than male patients.^45^, ^46^, ^47^ At the same time, it has been stated in previous studies that men with cancer have more sexual information needs than women with cancer.^50^, ^51^ Women have been passive in getting sexual information compared to men.^52^, ^53^ This may be due to cultural factors and embarrassment.^54^, ^55^

For the treatment and management of cancer, there are various treatments such as surgery, chemotherapy, and so forth.^56^ Worldwide, more than 60% of medical treatments focus on cancer.^57^ Nevertheless, as in this study and many other studies,^56^, ^58^ cancer patients usually use factors such as survival time and cost to choose the appropriate treatment. In the study of Tabriz et al.^28^ as in our study, financial needs were the most important needs of patients. The reason for this can be related to the weak insurance coverage in Iran. In the study of Khoshnazar et al.^59^ most patients believed that the insurance coverage in Iran was insufficient and did not meet their needs. While the side effects of different treatment methods are very important in choosing the appropriate treatment, sometimes not choosing the right treatment in time may lead to the patient's death.^60^ Therefore, education is necessary to empower patients and their deep understanding of treatment methods to promote correct decision‐making in Iran.

Diagnosing cancer can lead to feelings of panic, anxiety, depression, and hopelessness,^61^ as well as raising doubts about the patient's future and dramatically increasing the patient's spiritual needs. An examination of the participants' experiences in the present study showed that the spiritual realm was one of the “reasons for not meeting information needs.” Since the people of Iran are religious^27^ and religion becomes more apparent when people face a life crisis,^62^ instead of seeking information about the disease; however, Lim and Yi^63^ believe that after diagnosis, religion can be used as a psychosocial approach in religious patients. However, due to the limitations of our cross‐sectional study, no cause‐and‐effect relationship can be established. Our research team suggests further investigating the role of religion in the future.

One of the physicians' tasks is to empower cancer patients to perform self‐care behaviors. Also, some studies have shown that communicating with physicians and receiving clear answers are vital in reducing anxiety and increasing patient satisfaction with the provision of care.^64^, ^65^ While in the present study, the participating patients considered their treating physicians as one of the “reasons for not meeting the information needs” due to lack of time and incomprehensible answers. This difference can be attributed to physicians' performance due to social, economic, political and cultural issues in Iran.^26^

The findings of this study can provide many implications in clinical practice to health care professionals, health systems and policymakers so that necessary interventions can be planned and carried out based on the needs of patients. When deciding on treatment options, the cost factor must be considered because the patient's financial ability and insurance status will play an important role in the patient's treatment follow‐up. According to previous studies, most of these patients will always face financial problems.^66^, ^67^ For this reason, insurance services, financial support services and policies in the pharmaceutical industry should be considered. Salarvand et al.^68^ have stated that one of the goals of the health reform plan in Iran is to reduce patients' out‐of‐pocket payments and, as a result, increase their satisfaction.

Health care professionals, family and friends should provide psychological and social support for these patients. For this purpose, you can benefit from the knowledge and facilities of support groups for cancer patients at the community level. Mass media can also greatly impact patients' knowledge and reduce the stigma of suffering from this disease by creating culture and education and, as a result, improve cancer patients' quality of life. If the quality of life increases, surely patients will make more efforts for self‐management and self‐control of their disease, which will improve their health level.

This study has several limitations. Because qualitative studies are limited to a small number of participants and a specific environment, they have no claim for their generalizations and results. Therefore, the lack of generalizability of the findings of the present study can be considered a limitation. In this study, only patients' perceptions were analyzed. Therefore, our research team recommends that future research, using caregivers' and experts' opinions and views, provide a broader perspective on unmet information needs. Since all participants were selected from the training hospital environment, the perceptions and experiences of patients with unmet information needs in other settings were not recorded. Participation was limited to people fluent in Persian, and the findings may not reflect the perceptions of patients who are not fluent in the language. Finally, it can be stated that the most important limitation of this research is that some needs, such as the sexual needs of the patients, should be asked in the interviews, which was in contradiction with the Iranian culture.

## 5 CONCLUSION

The results of the present study show that when addressing the unmet information needs of cancer patients, the underlying causes of their unmet information needs should also be evaluated and considered. Patients may, for reasons such as shyness, refuse to express certain information needs, such as the need for sexual information, or, because of the imminence of death, seek spiritual information instead of information about the disease. Therefore, in countries with Muslim people, counseling and educating cancer patients to improve their knowledge by doctors and government organizations will lead to the empowerment of patients. It will also play a key role in the timely control and treatment of this disease.

## AUTHOR CONTRIBUTIONS

**Parasto Amiri**: Conceptualization; formal analysis; writing – original draft; writing – review & editing. **Ali Mohammadi**: Conceptualization; formal analysis; writing – original draft. **Kambiz Bahaadinbeigy**: Conceptualization; writing – original draft; writing–review & editing. **Behjat Kalantari Khandani**: Writing – review & editing. **Vahid Maazed**: Writing – review & editing.

## CONFLICT OF INTEREST

The authors declare no conflict of interest.

### ETHICS STATEMENT

This article was extracted from an independent research project performed at Kerman University of Medical Sciences without organizational support (code: IR.KMU.REC.1399.026).

### TRANSPARENCY STATEMENT

The lead author Kambiz Bahaadinbeigy affirms that this manuscript is an honest, accurate, and transparent account of the study being reported; that no important aspects of the study have been omitted; and that any discrepancies from the study as planned (and, if relevant, registered) have been explained.

## Supporting information

Supporting information.

## Data Availability

Our data or material may be available from the corresponding author or first author upon reasonable request.
