# Supplementary material for: Identifying unmet information needs of advanced cancer patients in Iran: An in‐depth qualitative study
Source: Health Sci Rep. 2022 Oct 29;5(6):e914. doi: 10.1002/hsr2.914 (PMC9617646; doi:10.1002/hsr2.914)
Supplement: Supplementary file 1 — Supporting information. [file HSR2-5-e914-s001.docx]

|  | **Standards for Reporting Qualitative Research (SRQR)*** |  |  |
| --- | --- | --- | --- |
|  | <http://www.equator-network.org/reporting-guidelines/srqr/> |  |  |
|  |  | **Page no** |  |
|  | |  |  |
|  | **Title and abstract** | |  |
|  | **Title** - Concise description of the nature and topic of the study. Identifying the study as qualitative or indicating the approach (e.g., ethnography, grounded theory) or data collection methods (e.g., interview, focus group) is recommended | The abstract indicates it is a qualitative evaluation study pg. 1 |  |
|  | **Abstract** - Summary of key elements of the study using the abstract format of the intended publication; typically includes background, purpose, methods, results, and conclusions | Semi-structured and in-depth interviews were conducted with selected patients to collect data. pg. 1 |  |
|  |  |  |  |
|  | |  |  |
|  | |  |  |
|  | **Introduction** | |  |
|  | **Problem formulation** - Description and significance of the problem/phenomenon studied; review of relevant theory and empirical work; problem statement | Have described background to the study, illustrating the importance and types of experience of patients pg. 1-2 |  |
|  | **Purpose or research questio**n - Purpose of the study and specific objectives or questions | The aim of the study is stated in the abstract pg. 1 and pg. 2 |  |
|  |  |  |  |
|  | |  |  |
|  | **Methods** | |  |
|  | **Qualitative approach and research paradigm** - Qualitative approach (e.g., ethnography, grounded theory, case study, phenomenology, narrative research) and guiding theory if appropriate; identifying the research paradigm (e.g., postpositivist, constructivist/ interpretivist) is also recommended; rationale** | The approach in which the study is situated is described in the Methods section pg. 3 |  |
|  | **Researcher characteristics and reflexivity** - Researchers’ characteristics that may influence the research, including personal attributes, qualifications/experience, relationship with participants, assumptions, and/or presuppositions; potential or actual interaction between researchers’ characteristics and the research questions, approach, methods, results, and/or transferability | We have addressed researcher reflexivity explicit in the Method pg. 3 |  |
|  | **Context** - Setting/site and salient contextual factors; rationale** | The setting is described on pg. 3-4 |  |
|  | **Sampling strategy** - How and why research participants, documents, or events were selected; criteria for deciding when no further sampling was necessary (e.g., sampling saturation); rationale** | The sampling strategy is addressed in the Methods pg. 3 |  |
|  | **Ethical issues pertaining to human subjects** - Documentation of approval by an appropriate ethics review board and participant consent, or explanation for lack thereof; other confidentiality and data security issues | Evidence of ethical approval is documented in Ethics pg. 5 |  |
|  | **Data collection methods** - Types of data collected; details of data collection procedures including (as appropriate) start and stop dates of data collection and analysis, iterative process, triangulation of sources/methods, and modification of procedures in response to evolving study findings; rationale** | The data collection methods is addressed in the Methods pg. 3 |  |
|  | **Data collection instruments and technologies** - Description of instruments (e.g., interview guides, questionnaires) and devices (e.g., audio recorders) used for data collection; if/how the instrument(s) changed over the course of the study | Data collection instruments is questionnaire is addressed in the Methods pg. 3 |  |
|  | **Units of study** - Number and relevant characteristics of participants, documents, or events included in the study; level of participation (could be reported in results) | The number and characteristics of the participants is provided on pg. 3 |  |
|  | **Data processing** - Methods for processing data prior to and during analysis, including transcription, data entry, data management and security, verification of data integrity, data coding, and anonymization/de-identification of excerpts | We have described the data processing in the Methods section pg. 3 |  |
|  | **Data analysis** - Process by which inferences, themes, etc., were identified and developed, including the researchers involved in data analysis; usually references a specific paradigm or approach; rationale** | The data analysis is processing in the Methods section pg. 3-4 |  |
|  | **Techniques to enhance trustworthiness** - Techniques to enhance trustworthiness and credibility of data analysis (e.g., member checking, audit trail, triangulation); rationale** | Have conducted a co-analysis of data with co-researchers. This is outlined in the Methods section (Step 2) pg. 5 |  |
|  |  |  |  |
|  | |  |  |
|  | **Results/findings** | |  |
|  | **Synthesis and interpretation** - Main findings (e.g., interpretations, inferences, and themes); might include development of a theory or model, or integration with prior research or theory | The main findings presented in the Results section pg. 5-13 |  |
|  | **Links to empirical data** - Evidence (e.g., quotes, field notes, text excerpts, photographs) to substantiate analytic findings | The evidence of the findings is linked to the use of quotations and field notes pg. 5-13 |  |
|  |  |  |  |
|  | |  |  |
|  | **Discussion** | |  |
|  | **Integration with prior work, implications, transferability, and contribution(s) to the field -** Short summary of main findings; explanation of how findings and conclusions connect to, support, elaborate on, or challenge conclusions of earlier scholarship; discussion of scope of application/generalizability; identification of unique contribution(s) to scholarship in a discipline or field | The Discussion summarises the main findings and discuss these in relation to existing literature in the dementia field pg. 13-15 |  |
|  | **Limitations** - Trustworthiness and limitations of findings | We have provided a Limitations section on pg. 15 |  |
|  | |  |  |
|  |  |  |  |
|  | **Other** | |  |
|  | **Conflicts of interest** - Potential sources of influence or perceived influence on study conduct and conclusions; how these were managed | No conflicts of interest |  |
|  | **Funding** - Sources of funding and other support; role of funders in data collection, interpretation, and reporting | We have provided a funding declaration and acknowledgements |  |
|  |  |  |  |
|  | *The authors created the SRQR by searching the literature to identify guidelines, reporting standards, and critical appraisal criteria for qualitative research; reviewing the reference lists of retrieved sources; and contacting experts to gain feedback. The SRQR aims to improve the transparency of all aspects of qualitative research by providing clear standards for reporting qualitative research. |  |  |
|  |  |  |  |
|  | **The rationale should briefly discuss the justification for choosing that theory, approach, method, or technique rather than other options available, the assumptions and limitations implicit in those choices, and how those choices influence study conclusions and transferability. As appropriate, the rationale for several items might be discussed together. |  |  |
|  |  |  |  |
|  | **Reference:** |  |  |
|  | O'Brien BC, Harris IB, Beckman TJ, Reed DA, Cook DA. **Standards for reporting qualitative research: a synthesis of recommendations.** *Academic Medicine*, Vol. 89, No. 9 / Sept 2014  DOI: 10.1097/ACM.0000000000000388 |  |  |
|  |  |  |  |
|  |  |  |  |
